# Redundancy Gains in Absolute Judgments of Loudness and Brightness

**DOI:** 10.1007/s42113-025-00236-w

**Published:** 2025-02-05

**Authors:** Robert C. G. Johansson, Rolf Ulrich

**Affiliations:** Fachbereich Psychologie, Eberhard Karls Universität Tübingen, Schleichstraße 4, 72076 Tübingen, Germany

**Keywords:** Intensity identification, Redundancy gain, Sequential analysis, Poisson model

## Abstract

People’s ability to discern the physical intensity level of visual and auditory events presented at the same time is investigated in a bimodal identification paradigm with stimulus redundancy. Two approaches to modeling redundancy gains in choice reaction time (RT) and response probability in this paradigm are advanced: first, a separate activation model where two sequential likelihood ratio tests (SLRTs) for exponentially distributed neural interarrival times operate on parallel channels, each capable to evoke a response, and second, a coactive model where the outputs of two SLRTs are superposed in a single processing channel to trigger a response. Although both models predict plausible error rates, the separate activation model accounts better for observed benchmarks in choice RT. However, a violation of the race model inequality at the smallest quantile for loud and soft bimodal stimuli hints that the separate activation model might nonetheless be untenable. These findings challenge both parallel and coactive processing accounts of performance in intensity identification tasks with redundant auditory-visual stimuli.

When people monitor multiple sensory channels for a target, they generally respond more quickly when a stimulus appears in several channels simultaneously compared to a single channel alone (Raab, 1962; Hershenson, 1962; Todd, 1912). For example, they might be instructed to respond to a sound, a light, or both as rapidly as possible using the same manual action. When sound and light appear together, they are redundant because each stimulus component independently conveys all the information necessary to perform the task. Hence, this phenomenon is termed a *redundancy gain*. Redundancy gains in both response time (RT) and response probability have been documented across a wide range of cognitive tasks, stimulus attributes, and sensory modalities (e.g., Mordkoff and Yantis, 1993; Patching and Quinlan, 2004; Blurton et al., 2014; Grice et al., 1984; Miller, 1986; Minakata and Gondan, 2019; Colonius et al., 2017; Fiedler et al., 2011). The present research investigated whether stimulus redundancy facilitates performance in a bimodal intensity identification paradigm with auditory and visual stimuli.

## Stimulus Redundancy in Multidimensional Identification Tasks

In the identification paradigm, people are required to label stimuli based on basic physical attributes, such as visual luminance or acoustic frequency (Wever & Zener, 1928; Garner, 1953; Engen & Pfaffmann, 1959; Warden & Rowley, 1929; Wolfle, 1937; Durlach & Braida, 1969; Pollack, 1952). For instance, participants might be presented with a visual stimulus of either 10, 20, 30, or 40 cd/m$^2$ luminous intensity and tasked with identifying the stimulus by choosing one of of four possible responses. Because there is a distinctive 1-to-1 mapping between stimulus value and response class in this task, identification paradigms are also known as “absolute judgment” paradigms. Admirable reviews of this topic are available elsewhere (e.g., Luce, 1986; Stewart et al., 2005; Shiffrin and Nosofsky, 1994; Vickers, 1979; Miller, 1956). Pertinent to the present study is the impact of informational redundancy on performance in identification tasks with multidimensional stimuli, as briefly reviewed next.

A classic study by Beebe-Center et al. (1955) examined the effects of stimulus redundancy on absolute judgments of primary tastants saline and sucrose. Participants were tasked with identifying solutions based on the molar concentration of the solute, for example, to determine whether a solution contained 0.3, 1, 4.8, or 34.7 g of sugar per unit solvent. Solutions were either simple (containing only salt *or* sugar) or compound (containing both salt *and* sugar). Compound solutions were redundant in that the least salty compound was also the least sweet, the saltiest compound was also the sweetest, and so forth. Absolute judgments of compound solutions were consistently more accurate than judgments of simple solutions, suggesting that people were able to use both tastant sensory cues to aid performance. Comparable redundancy gains in response probability have been demonstrated for identification tasks with simple tones varying in pitch and loudness (Pollack & Ficks, 1954; Fulgosi et al., 1975), lights varying in hue and brightness (Flowers, 1974), and cutaneous electric stimuli varying in duration and current amplitude (Hawkes, 1962).

## Integrated Processing vs. Parallel Racing

Debates have sparked regarding whether redundancy gains indicate integrated neural processing of multiple information sources or reflect parallel processing of the redundant stimulus components on separate channels (Raab, 1962; Miller, 1982; Mulligan & Shaw, 1980; Fidell, 1970). Raab (1962) demonstrated that a horse race model can explain performance gains (shorter RTs) for redundant stimuli without positing any interaction between sensory channels. The horse race model conceives of the processing of redundant information sources as a race between two or more evidence accrual processes on parallel sensory channels, each striving to reach a decision threshold. The first accrual process to reach this threshold determines observed RT on that trial by triggering a manual response. When the distributions of processing times in the two channels overlap, the expected value of their minimum is less than the average processing time of either channel considered in isolation: a mechanism termed “statistical facilitation.” Hence, the race model cements a plausible neural account of redundancy gains in RTs, which does not assume sensory channel interactions of any sort.

A critical evaluation of Raab (1962)’s approach was introduced by Miller (1982), who derived an upper limit to the magnitude of statistical facilitation predicted by horse race models. Miller articulated the notion that no response to redundant stimuli can be faster than the quickest response elicited by non-redundant stimuli if processing proceeds in parallel. This relation is encapsulated by the race model inequality

1 $$\begin{aligned} F_{AV}(t) \le \min (F_A(t) + F_V(t),1) \end{aligned}$$

where $F_A(t) = P(RT_A \le t)$ and $F_V(t) = P(RT_V \le t)$ represent the cumulative density functions (CDFs) of the RTs to auditory and visual unimodal stimuli, and $F_{AV}(t)=P(RT_{AV} \le t)$ is the CDF of RTs on bimodal trials. Hence, the right side of Inequality Eq. 1 represents the theoretically inferred CDF for RTs to redundant stimuli when processing events on parallel channels race to evoke a behavioral response. The race model inequality serves as a criterion to assess whether observed redundancy gains can be attributed solely to parallel racing on separate channels or whether additional integrative neural mechanisms might be involved. Multiple studies have demonstrated that choice RTs from decision tasks can violate the upper bound expressed by Inequality Eq. 1 (e.g., Blurton et al., 2014; Hecht et al., 2008; Suied et al., 2009; Zehetleitner et al., 2015; Miller, 1991). A violated race model inequality is typically interpreted as evidence for a coactive processing architecture, where information from multiple sensory channels interacts synergistically to influence behavioral responses (Miller, 2016).

## Redundant Intensity Identification

A go/no-go analogue of the intensity identification task with redundant auditory-visual stimuli was implemented by Minakata and Gondan (2019). They found that Inequality Eq. 1 was violated when loud tones and bright lights signaled go-trials, but not when soft tones and dim lights constituted the go-stimuli. As noted by Minakata and Gondan, this pattern of coactivation is particularly noteworthy because it contradicts the inverse effectiveness principle of multisensory integration (e.g., Stein and Stanford, 2008). Inverse effectiveness posits that redundancy gains should be most evident for faint stimuli. For example, multisensory neurons in the superior colliculus of the mammalian midbrain exhibit substantial bimodal gains in firing rate for concurrent weak auditory and visual stimuli, but firing rate increments for strong auditory-visual stimuli are more modest (Wallace & Stein, 1998; Meredith & Stein, 1983, 1986).

Minakata and Gondan’s 2019 findings raise questions about the generality of the inverse effectiveness principle, suggesting it may not apply to intensity identification paradigms involving redundant auditory-visual stimuli. This would suggest a theoretically significant dissociation between multisensory processing gains at the neural and behavioral levels (topic reviewed in Otto et al., 2013). Alternatively, these data might reflect performance characteristics specific to go/no-go paradigms rather than intrinsic aspects of stimulus processing. For instance, Ulrich et al. (1999) have suggested that the response initiation mechanism might differ in meaningful ways between choice RT and go/no-go tasks due to differences in response probability between the two tasks. In light of this, we aimed to adapt the go/no-go intensity identification task of Minakata and Gondan (2019) to a standard choice RT paradigm with two response options. To further enhance the insight gained from this experiment, we also developed and tested two computational models of multisensory redundancy gains in behavioral performance for this task. Both models are elaborations of the Poisson-SLRT model of two-choice intensity identification (Johansson & Ulrich, 2025), here adapted to paradigms involving stimulus redundancy as outlined in the following sections.

### Sequential Intensity Identification with Poisson-SLRT

The Poisson-SLRT model for two-choice intensity identification tasks is predicated on the principle that stimulus intensity is represented by the rate of neural spike generation within a sensory pathway. Spike generation follows a stationary Poisson process where the rate parameter $\lambda$ is a monotonic increasing function of stimulus intensity. To determine the intensity of the stimulus, each neural spike is timed sequentially as follows: Let $T_i$ denote the observation time of the $i^{th}$ neural spike $(i=1,2,\dots )$ following stimulus onset at $T_0=0$. The interarrival time (IAT) between successive spikes is then the independently and identically distributed exponential random variable $X_i=T_i-T_{i-1}$. Upon presentation of the $j^{th}$ stimulus $S_j$ with the associated rate $\lambda _j$ ($j=w,s$), the expected value of the IATs between successive neural spikes is $E[X|S_j] = 1/ \lambda _j = \theta _j$. The subscripts *w* and *s* should be read as “weak” and “strong,” respectively. Consequently, $S_s$ (strong stimulus) is associated with a faster rate of spike generation and shorter expected IATs than $S_w$ (weak stimulus). To evaluate the evidence provided by the $i^{th}$ neural spike latency $X_i$ given $\theta _s$ and $\theta _w$, the negative log-likelihood ratio serves as the metric, so that

2 $$\begin{aligned} \Lambda _i= &  - \log \left[ \frac{L(\theta _s|X = x_i)}{L(\theta _w|X = x_i)} \right] \end{aligned}$$

3 $$\begin{aligned}= &  - \log \left[ \frac{ \frac{1}{\theta _s} \exp (-x_i/\theta _s) }{\frac{1}{\theta _w} \exp (-x_i/\theta _w) } \right] \end{aligned}$$

4 $$\begin{aligned}= &  - \log \left( \frac{\theta _s}{\theta _w} \right) + \frac{\theta _s - \theta _w}{\theta _s \theta _w} \cdot X_i \end{aligned}$$

The logarithmic operator in Eq. 2 assures that $\Lambda _i$ is positive whenever $X_i$ favors $S_s$, and negative when $S_w$ is favored.

Next, we assume a retention unit $\Sigma$ which holds the gathered evidence in store as it accumulates over the course of an experimental trial, always departing from the same starting point at $\Sigma _0 = 0$. Following the arrival of the $i^{th}$ neural spike at time $T_i$, the retention state is updated to

5 $$\begin{aligned} \Sigma _i = \Sigma _{i-1} + \Lambda _i. \end{aligned}$$

At stimulus onset, $\Sigma$ initiates a random walk over the real line, with discontinuities occurring at each spike time $T_i$. This random walk will tend towards the positive domain when the stimulus is strong, and the negative domain when the stimulus is weak. The stopping rule for stimulus sampling is derived from the theory of sequential analysis (Wald, 1945).

Following Wald, we can determine two critical absorption barriers, *A* and *B*, such that the type I and type II error rates of the test procedure do not exceed some desired proportions $\alpha$ and $\beta$

6 $$\begin{aligned} A (\alpha , \beta ) \approx \log \frac{1-\beta }{\alpha } \end{aligned}$$

and

7 $$\begin{aligned} B (\alpha , \beta ) \approx \log \frac{\beta }{1-\alpha } \end{aligned}$$

We can directly estimate $\alpha$ and $\beta$ based on empirical response probabilities. The barriers *A* and *B* establish a continue-sampling region, so that the test procedure perpetuates as long as $A> \Sigma _i > B$. If $\Sigma _i$ is absorbed at *A*, a response in favor of $S_s$ is emitted. Conversely, a response in favor of $S_w$ is emitted if $\Sigma _i$ is absorbed at *B*. The total decision time *DT* for stimulus identification can therefore be expressed as

8 $$\begin{aligned} DT = \min (T_i\!: \, \Sigma _i \ge A \vee \Sigma _i \le B), \end{aligned}$$

The predicted response time is given by $RT = DT + M$. Here, *M* is a random normal deviate with mean $\mu$ and standard deviation $\sigma$ that accounts for non-decision latency components such as transmission and motor delays.

#### Separate Activation Model

**Fig. 1:**
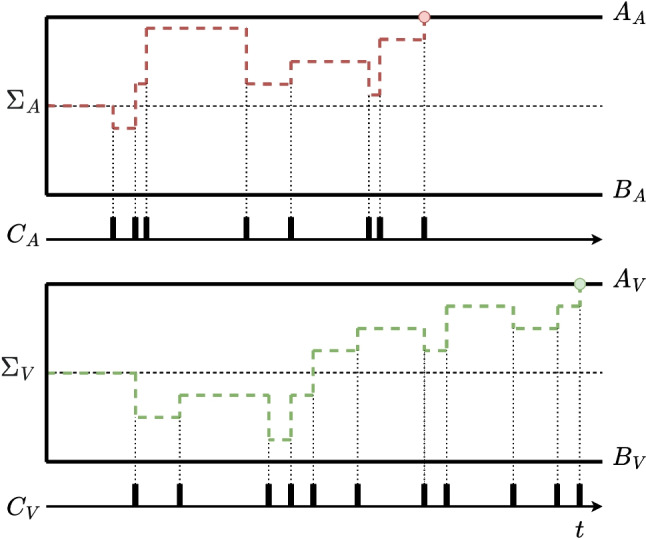
Schematic illustration of the separate activation Poisson-SLRT model. Neural IATs registered on auditory ($C_A$) and visual ($C_V$) input channels are evaluated by separate SLRT procedures which “race” towards absorption on bimodal trials. The first procedure to terminate determines the decision latency. In this illustration, auditory identification time (red circle) is shorter than visual identification time (green circle). Therefore, on this trial, the observed RT is determined by auditory identification time

We now consider a two-choice identification task with auditory and visual stimuli, where each stimulus component (visual or auditory) can be either “weak” (dim or soft) or “strong” (bright or loud). Critically, the two stimulus components are perfectly correlated when they appear together: a soft tone always accompanies a dim light, and a loud tone always accompanies a bright light. This correlation renders the bimodal stimulus redundant. The separate activation model posits that neural IATs from auditory and visual channels are processed by two independent SLRTs operating in parallel. During bimodal trials, each SLRT evaluates the intensity level of its respective stimulus component without interaction from the other. The decision process is self-terminating because identifying the intensity of either the auditory or visual component is sufficient to make a response. For ease of exposition, it is helpful to consider the two evidence accrual mechanisms as continuous functions of time: Let $\Sigma _A(t)$ and $\Sigma _V(t)$ denote the time-dependent states of the retention units on auditory and visual channels at time *t*. This yields the following expression for decision time:

9 $$\begin{aligned} DT = \min (t\!: \, \Sigma _A(t)&\ge A_A \vee \Sigma _A(t) \le B_A \vee \Sigma _V(t)\nonumber \\ &\ge A_V \vee \Sigma _V(t) \le B_V ), \end{aligned}$$

where $A_A$ and $B_A$ are the absorption barriers for the auditory channel, and $A_V$ and $B_V$ are the barriers for the visual channel. In other words, the response class (weak or strong) and the response time (RT) are both determined by the first SLRT that reaches an absorption barrier. This model architecture is depicted schematically in Fig. 1.

**Fig. 2:**
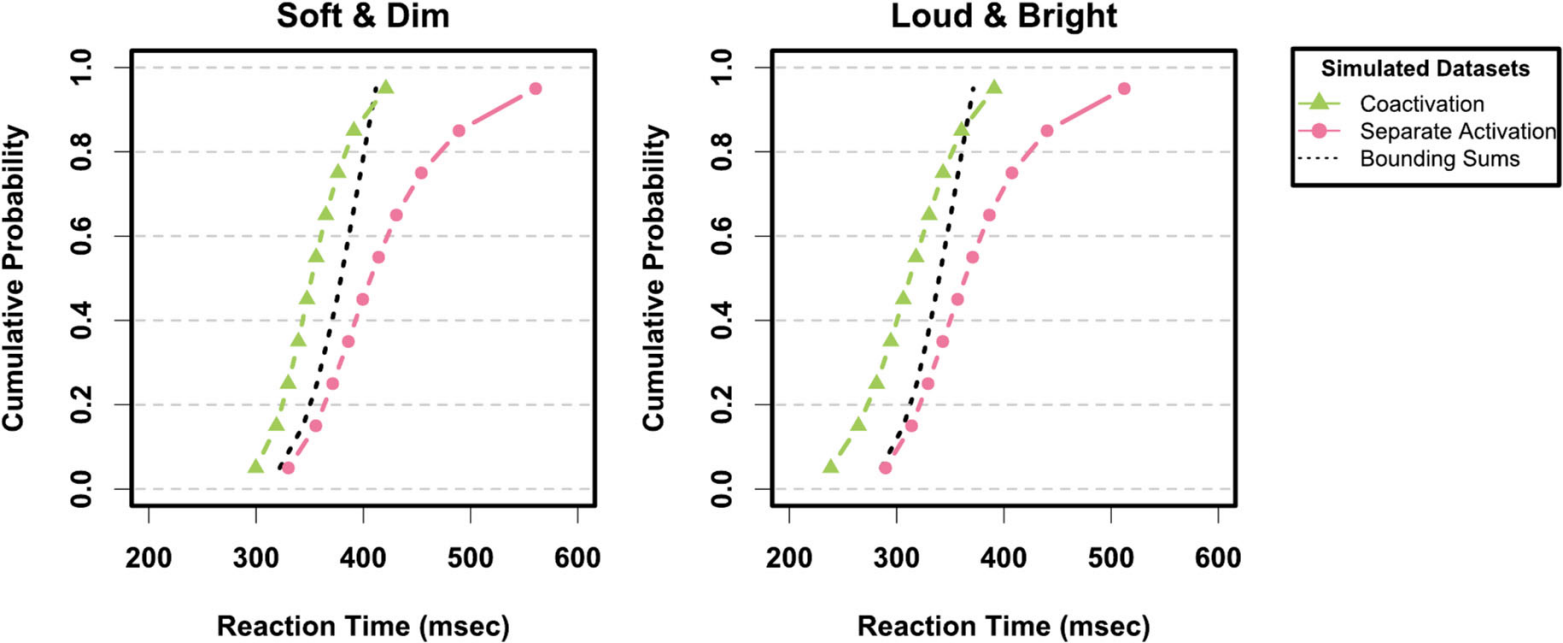
Simulation results depicting RTs on bimodal trials as predicted by the coactive model (pink triangles) and the separate activation model (green circles), contrasted with the bounding sums for RTs on unimodal trials predicted by the Poisson-SLRT model (black dashes). These data are conveyed separately for weak (left panel) and strong intensity stimuli (right panel). Parameters for this simulation were $\lambda _{A,w}=\lambda _{V,w}=100$ Hz, $\lambda _{A,s}=\lambda _{V,s}=200$ Hz, $M_A=300$ ms, $M_V=320$ ms, $\sigma _A=\sigma _V=30$, and desired error proportions of $5\%$

The separate activation model embodies the assumptions of a model class characterized by unlimited capacity independent parallel processing with an OR-decision rule (e.g., Wenger and Townsend, 2000; Townsend and Ashby, 1983; Townsend and Nozawa, 1995). It assumes that visual and auditory stimuli are identified at the same time on separate channels (parallel processing), and that decision times for visual stimuli remain unaffected by whether auditory stimuli are present or absent and vice versa (context independence, e.g., Luce (1986); Colonius (1990)). Finally, because stimulus processing is self-terminating (OR-decision rule), the separate activation model functions like a standard race model and predicts that Inequality Eq. 1 should not be violated for any value of $t > 0$ as depicted in Fig. 2.

#### Coactive Model

The coactive extension of the Poisson-SLRT model proposes an integrative processing pathway where evidence accumulation from auditory and visual channels are combined during bimodal trials. When a bimodal stimulus is presented, a new superposed accrual processes $\Sigma _{AV}(t)$ can be construed by combining the outputs of the two SLRT procedures, so that

10 $$\begin{aligned} \Sigma _{AV}(t) = \Sigma _{A}(t) + \Sigma _{V}(t) \end{aligned}$$

**Fig. 3:**
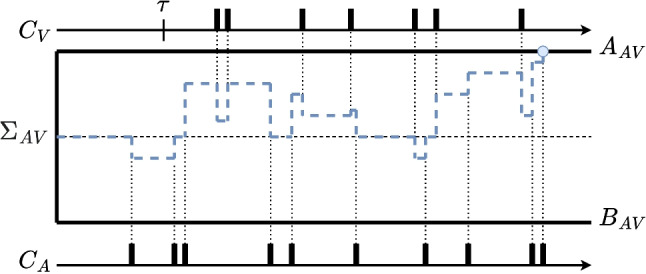
Schematic illustration of the coactive Poisson-SLRT model. Neural IATs registered on auditory ($C_A$) and visual ($C_V$) input channels are evaluated by an integrated test procedure $\Sigma _{AV}$ which pools the gathered sensory evidence across modalities. In this example, visual processing is delayed relative to auditory processing by $\tau$ units time

Equation 10 describes a random walk step-function with discontinuities at each visual *and* auditory spike time, as schematically illustrated in Fig. 3. The absorption barriers for the superposed accrual process $\Sigma _{AV}(t)$ are again estimated based on empirical confusion probabilities for bimodal trials, following Eqs. 6 and 7.

Importantly, we must consider the possibility that the non-decision times for auditory ($M_A$) and visual ($M_V$) processing channels might differ. Let $\tau = M_V - M_A$ denote the time lag between the onsets of the decision processes on visual and auditory channels. When the $j^{th}$ bimodal stimulus (again, $j=w,s$) is presented, the superposed process will proceed at a rate $\lambda _{AV,j}$ which can be expressed as

11 $$\begin{aligned} \lambda _{AV,j} = {\left\{ \begin{array}{ll} \lambda _{A,j}, & \text {if } t< \tau \wedge M_V > M_A \ \\ \lambda _{V,j}, & \text {if } t< -\tau \wedge M_V < M_A \ \\ \lambda _{A,j} + \lambda _{V,j}, & \text {if } t \ge |\tau | \end{array}\right. } \end{aligned}$$

where $\lambda _{A,j}$ and $\lambda _{V,j}$ are the transduction rates estimated from unimodal auditory and visual trials, respectively. Because the two bimodal stimulus components are perfectly correlated, $\Sigma _{A}$ and $\Sigma _{V}$ will tend toward the same absorption barrier on those trials when both auditory and visual signals are presented. Consequently, we should expect $\Sigma _{AV}$ to reach the absorption barrier more quickly on average than either $\Sigma _{A}$ or $\Sigma _{V}$, provided the absorption barriers for all three processes are of similar magnitude. This is also demonstrated in Fig. 2 with simulated data. Notably, the coactive model predicts that the race model inequality will be violated for some values of $t > 0$.

## Experiment

The aim of this experiment was to shed new light on how human observers discern the intensity level of visual and auditory events presented at the same time. To this end, we implemented a two-choice intensity identification task featuring soft or loud tones (auditory condition), dim or bright lights (visual condition), and a combination of both (bimodal condition). Participants were tasked to determine the identity of the stimulus (weak or strong) by pressing one out of two possible buttons. Visual and auditory stimulus components were redundant on bimodal trial types, so the dim light always appeared with the soft tone, and the bright light always accompanied the loud tone. This ensured that identifying the intensity level of either bimodal stimulus component was sufficient to provide a response. In this regard, the experiment mirrored the go/no-go task implemented by Minakata and Gondan (2019), here adapted to the domain of binary choice.

Our specific goals were threefold: firstly, to determine whether choice RTs to redundant auditory-visual stimuli in the bimodal intensity identification paradigm violate the race model bound (Inequality Eq. 1), and secondly, to fit the Poisson-SLRT model to the RT distributions for unimodal trial types to abstract parameters for further modeling efforts. Our third and primary goal was to evaluate whether the separate activation model or the coactive model better accounts for human performance in this task on the basis of parameters estimated in Step 2.

### Methods

#### Participants

Thirty participants (6 males) with a mean age of 26 years (age range, 20–40 years) were recruited from the student pool at Tübingen University. Participants received compensation in the form of either 12€ or mandatory course credit for their participation in a single, 45-min session. Everyone reported normal- or corrected-to-normal seeing and normal hearing and provided informed consent. Sample size was determined based on available resources for testing.

#### Procedure

Participants were initially presented with written instructions on the monitor which explained the aim of the task (“Determine whether the stimulus is weak or strong”). The instructions emphasized the importance of responding both quickly and accurately. Participants then completed six experimental blocks consisting of 96 trials each, always preceded by practice blocks of 24 trials. These practice blocks were designed to ensure participants were familiar with the current stimulus–response mapping, which changed pseudo-randomly between blocks (e.g., from weak-left and strong-right to weak-right and strong-left). Thus, each session included three practice blocks and three experimental blocks for each stimulus–response mapping. Visual, auditory, and bimodal trials were intermixed within blocks. Participants were allowed to rest between each block.

Each trial began with the appearance of a faint fixation cross in the center of the monitor for 500 ms, followed by a 500-ms foreperiod. Subsequently, a stimulus appeared for 1000 ms or until a response was registered. If the response was incorrect, the German word for error (“Fehler”) was displayed in a red font in the center of the monitor for 500 ms. If the response was correct, a 500-ms blank screen served as the intertrial interval. The stimulus was a patch of light, a sinusoidal waveform tone, or both, with each component having one of two possible intensity levels: the light could be dim or bright, and the tone could be soft or loud.

#### Stimuli and Apparatus

Stimulus presentation and response recording were managed by an Esprimo P956/E90+ microcomputer (Fujitsu Limited, Tokyo) running a PsychoPy script (Peirce et al., 2019) on a 64-bit Windows 10 OS. Visual stimuli were displayed on a 24.1-inch FlexScan EV2495 LCD monitor (EIZO Corporation, Hakusan) placed 57 cm in front of the viewer. Viewing distance was controlled with a chinrest. The monitor had a resolution of $1900 \times 1200$ pixels, and the refresh rate was 60 Hz. Background illumination was held constant at 0.3 cd/m$^2$ throughout the experiment. Visual stimuli were square patches of light measuring 1.5$^\circ$ $\times$ 1.5$^\circ$ visual angle presented for 1000 ms in the center of the monitor. The luminous intensity of dim and bright visual stimuli was 2 and 5 cd/m$^2$, respectively. Luminance was measured with a P-9201-TF photometer (Gigahertz Optik, Türkenfeld). Auditory stimuli were pure sinewaves at an acoustic frequency of 220 Hz, delivered binaurally through stereo speakers (PowerMax 80/2, TEAC Corporation, Tokyo) which flanked the monitor at 50 cm distance from the center. The soft tone was set at 60 decibel sound pressure level (A-weighted; dB(A) SPL), and the loud tone at 80 dB(A) SPL. Loudness level was measured with a CEL-275 sonometer (Casella CEL Instruments Ltd., Hitchin, UK). Responses were captured using a custom two-key response box interfaced via the computer’s parallel port. The average stimulus onset asynchrony of visual and auditory stimuli was 0.53 ms (SD = 1.14 ms), as verified with external chronometry using a BlackBox Toolkit (Version 2; Plant (2016))^1^ The experiment was conducted in a sound- and light-attenuated booth.

#### Data Analysis

Data analysis proceeded in four successive steps: First, the data was inspected for outlier RTs shorter than 200 ms (0.4%) or longer than 1750 ms (0%) which were classified as anticipations and misses, respectively. Anticipations and misses were excluded from further data analysis. Individual subjects with average accuracy rates below 75$\%$ were also excluded from the analysis (no cases). Then, error rates were entered into a 3 $\times$ 2 repeated measures ANOVA to assess the main and interaction effects of stimulus modality (auditory, visual, or bimodal) and stimulus intensity (weak or strong). Incorrect responses were removed from the analysis of RTs. Mean RTs were similarly entered into a 3 $\times$ 2 repeated measures ANOVA to test main and interaction effects of stimulus modality and stimulus intensity. The criterion threshold of statistical significance for these tests was $\alpha =.05$.

For the second step of data analysis, CDFs and bounding sums of the RTs pertinent for testing the race model inequality were computed across 10 quantiles (0.05, 0.15, ..., 0.95) for each participant individually following the procedures outlined by Ulrich et al. (2007). The race model inequality was then tested across each quantile using a permuted *t*-test with a $\alpha =.05$ criterion threshold of evidential strength as suggested by Gondan and Minakata (2015).

**Fig. 4:**
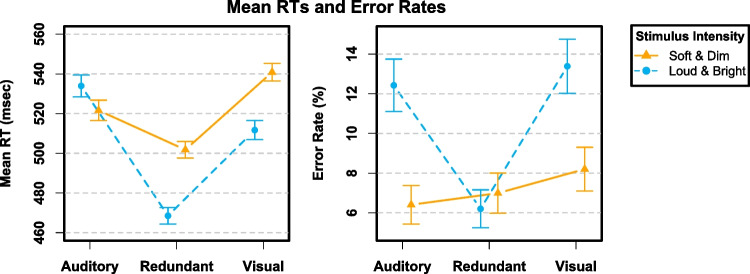
Experimental results conveyed in terms of mean RTs (left panel) and error rates (right panel). Weak intensity stimuli (soft tones and dim lights) are depicted as connected orange triangles, and strong intensity stimuli (loud tones and bright lights) are depicted as dashed blue circles. Error bars represent the within-subjects confidence intervals (95% CIs; Loftus and Masson, 1994) with the correction suggested by Cousineau (2005)

The third data analysis step entailed fitting the Poisson-SLRT model to the distributions of correct RTs from unimodal trial types. To this end, four group-level CDFs (2 modalities $\times$ 2 intensity levels) were constructed for correct RTs on unimodal trials across ten quantiles (0.05, 0.15, ..., 0.95) following Ratcliff (1979). Then, the Poisson-SLRT model was fitted separately to auditory and visual group-level CDFs for RTs using a downhill simplex routine (Nelder & Mead, 1965) with 10,000 sampled RTs per iteration. The fitting routine sought to minimize the aggregated $\chi ^2$ discrepancy between predicted and observed RT quantiles as given by

12 $$\begin{aligned} \chi ^2 = \sum _j \sum _q \frac{(O_{j,q}-E_{j,q})^2}{E_{j,q}} \end{aligned}$$

where $O_{j,q}$ and $E_{j,q}$ represent the observed and expected values of the $q^{th}$ quantile ($q=1,2,\dots ,10$) of the $j^{th}$ RT distribution ($j=w,s$, indicating “weak” or “strong”). Four free parameters were necessary for fitting the data for each modality: two rate parameters for the auditory trials ($\lambda _{A,w}$ and $\lambda _{A,s}$) and an auditory non-decision time defined by $\mu _A$ and $\sigma _A$. Two rate parameters ($\lambda _{V,w}$ and $\lambda _{V,s}$) were similarly estimated for the visual trials in addition to residual latency parameters $\mu _V$ and $\sigma _V$. Error RT CDFs were not fitted as some participants made very few errors.

In the fourth and final step of data analysis, both candidate extensions of the Poisson-SLRT model for intensity identification tasks with stimulus redundancy were fitted to the RT CDFs from bimodal trials. Both the separate activation model and the coactive model were fitted using the parameters estimated in the third step of data analysis. Consequently, no additional free parameters were required to evaluate model fit. The reported $\chi ^2$-values for the separate activation model and the coactive model are, therefore, strictly comparable. All data and analysis code are available via the OSF.^2^

### Results and Discussion

The analysis of error rates revealed main effects of stimulus intensity ($F(1,29)=20.93$, $p<.05$, $\eta _G^2=.056$) and stimulus modality ($F(2,58)=8.91$, $p<.05$, $\eta _G^2=.057$), and an intensity $\times$ modality interaction ($F(2,58)=6.07$, $p<.05$, $\eta _G^2=.042$). This pattern also held for mean RTs: main effects of stimulus intensity ($F(1,29)=27.55$, $p<.05$, $\eta _G^2=.012$) and stimulus modality ($F(2,58)=32.61$, $p<.05$, $\eta _G^2=.062$) were evident in addition to an intensity $\times$ modality interaction ($F(2,58)=30.22$, $p<.05$, $\eta _G^2=.017$). Mean RTs and error rates are illustrated in Fig. 4.

Pairwise contrasts of mean RTs revealed that bright visual stimuli were identified 28 ms faster than dim visual stimuli ($t(29)=6.11$, $p<.05$, $95\%$ CI $(19,\ 39)$). Loud auditory stimuli were on the other hand identified 11 ms slower than soft auditory stimuli ($t(29)=2.29$, $p<.05$, $95\%$ CI $(1,\ 22)$). Strong intensity bimodal stimuli were identified 32 ms faster than weak intensity bimodal stimuli ($t(29)=6.88$, $p<.05$, $95\%$ CI = $(23,\ 43)$). The redundancy gain in task accuracy on bimodal trials was 3.5 $\%$ ($t(29) = 5.85$, $p <.05$, $95 \%$ CI $(2.3,\ 4.7)$). For mean RTs, the redundacy gain was 42 ms ($t(29)=15.67$, $p<.05$, $95\%$ CI $(37,\ 48)$).

#### Race Model Inequality

**Fig. 5:**
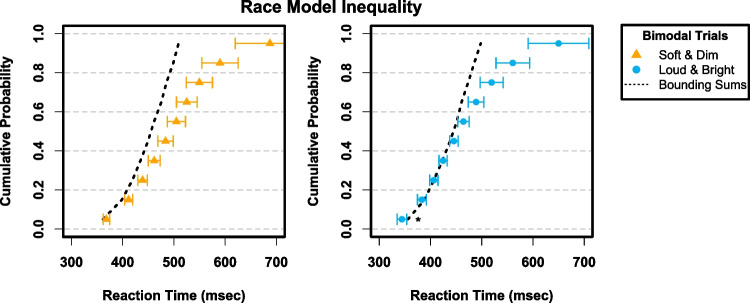
Experimental results conveyed in terms of the RT CDFs from bimodal trial types, separately for weak (orange triangles; left panel) and strong (blue circles; right panel) bimodal stimuli. These data are contrasted with theoretical bounding sums predicted by the horse race model (black dashed lines). The asterisk (*) in the right panel highlights a violation of Inequality 1 at the smallest (.05) quantile point. Error bars denote the 95% CI of the permuted difference score for each quantile

Contrasts of RT CDFs from bimodal trials against the theoretical bounding sums predicted by a parallel processing architecture revealed that the race model inequality was not violated for weak (soft and dim) bimodal stimuli (all *p*’s >.05). But for strong (bright and loud) bimodal stimuli, the race model inequality was violated by 11 ms at the smallest (.05) quantile point ($t(29) = 2.46$, $p <.05$). These results are visualized in Fig. 5.

#### Poisson-SLRT Model

**Fig. 6:**
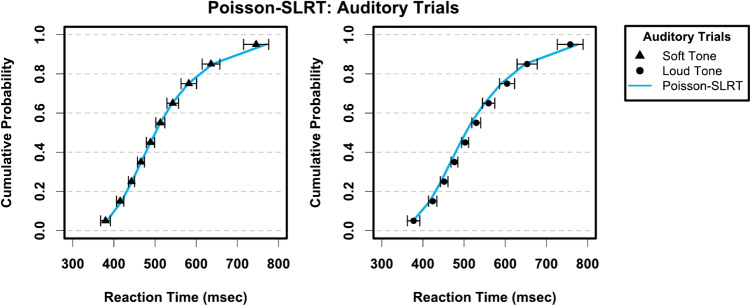
Unimodal trial type RT CDFs conveyed separately for soft (black triangles; left panel) and loud (lower panels) auditory stimuli. Predictions by the Poisson-SLRT model are denoted by the blue line segment. Error bars denote the within-subjects 95% CIs

For the visual condition, the rate parameters were $\hat{\lambda }_{V,w}=177$ Hz and $\hat{\lambda }_{V,s}=260$ Hz. Non-decision time parameters were $\hat{\mu }_{V}=400$ ms and $\hat{\sigma }_{V}=45$ yielding a fit of $\chi ^2=2.24$. Predicted error proportions for the dim and bright stimulus were 7.6% and 10.0%, which corresponded fairly well with the observed proportions (8.2% and 13.4%, respectively). The fit of the Poisson-SLRT model to correct RT CDFs from the visual condition is depicted in Fig. 6.

**Fig. 7:**
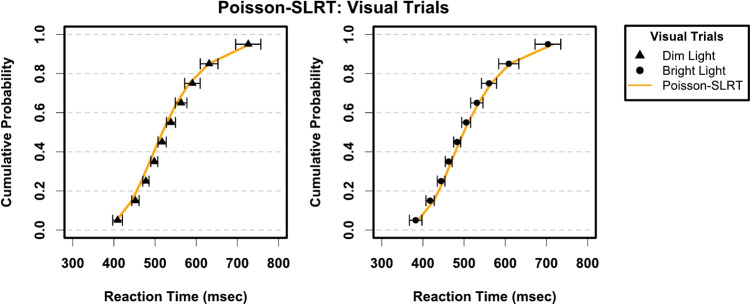
Unimodal trial type RT CDFs conveyed separately for dim (black triangles; left panel) and bright (lower panels) visual stimuli. Predictions of the Poisson-SLRT model are denoted by the orange line segment. Error bars denote the within-subjects 95% CIs

For the auditory condition, the estimated rate parameters were $\hat{\lambda }_{A,w}=411$ Hz and $\hat{\lambda }_{A,s}=516$ Hz. Non-decision time parameters $\hat{\mu }_{A}=370$ ms and $\hat{\sigma }_{A}=45$ yielded a best fit of $\chi ^2=2.84$. Predicted error proportions for the soft and loud stimulus were 5.9% and 10.1%, again fairly close to observed proportions (6.4% and 12.4%, respectively.) The fit of the Poisson-SLRT model to correct RT CDFs from the auditory condition is depicted in Fig. 7.

#### Separate Activation Model

**Fig. 8:**
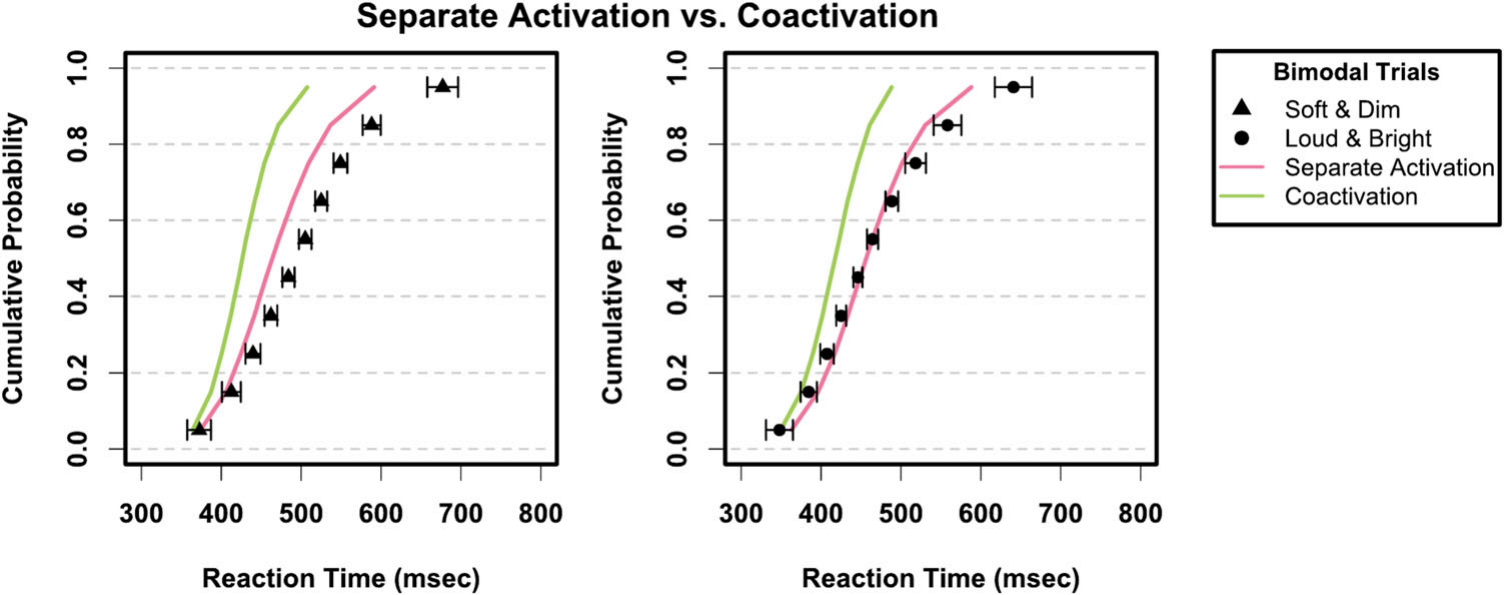
CDFs of the choice RT distributions as simulated with the separate activation model (purple line segment) and the coactive model (green line segment) contrasted with empirical choice RT CDFs. These data are depicted separately for soft and dim (black triangles; left panel) and loud and bright (black circles; right panel) redundant bimodal stimuli. Error bars denote the within-subjects 95% CIs

The separate activation model positing independent parallel processing of visual and auditory information sources yielded an aggregated distributional RT fit of $\chi ^2=33.90$. Predicted error proportions for the weak and strong bimodal stimuli were 7.1% and 9.6%, in decent agreement with observed error proportions (7.0% and 6.2%, respectively). Figure 8 illustrates the fit of the separate activation model to the RT CDFs from bimodal trials.

#### Coactive Model

The coactive model positing joint identification of visual and auditory stimulus intensity levels yielded a distributional fit $\chi ^2=203.21$, considerably worse than the separate activation model. Predicted error proportions for the weak and the strong bimodal stimuli were 10.5% and 1.7%, in acceptable agreement with empirical error proportions. The fit of the coactive model to the RT CDFs from bimodal trials is similarly illustrated in Fig. 8.

## General Discussion

The aim of this research was to characterize how people process concurrent visual and auditory events in a bimodal intensity identification paradigm with stimulus redundancy. Specifically, we investigated whether redundancy gains in choice RT were evident, and if so, whether their magnitude exceeded the predictions of a horse race model positing parallel processing of auditory and visual information sources on separate channels. To this end, we subjected RTs from the bimodal type trials to distributional contrasts to assess whether observed redundancy gains violated the race model inequality. Additionally, we tested two computational models of behavioral performance in this task: a separate activation model and a coactive model embodying the principles of parallel processing and integrated processing, respectively. As the separate activation model and coactive model predict different magnitudes of redundancy gains in RT for redundant auditory and visual stimuli, we assessed their ability to account for RT CDFs from bimodal trials in this task.

First, redundancy gains in behavioral performance for bimodal stimuli were evident in both choice RT and task accuracy. Distributional contrasts with the upper bound of Inequality Eq. 1 showed that the race model inequality was violated for strong bimodal stimuli, but not for weak bimodal stimuli. It is noteworthy that this differential pattern of coactivation for weak and strong stimuli corroborates the data from the go/no-go task reported by Minakata and Gondan (2019). To reiterate, Minakata and Gondan observed that evidence for coactivation was unique to strong intensity bimodal stimuli: only the CDFs for RTs from go-trials signaled by loud tones and bright lights violated the race model inequality. Our conceptual replication of these intriguing results in a standard choice RT paradigm strengthens the notion that human performance in intensity identification tasks with redundant auditory-visual stimuli is somehow at odds with the inverse effectiveness principle of multisensory integration. It also poses a challenge for models positing parallel stimulus processing in this task.

Nonetheless, the separate activation model demonstrated a satisfactory fit to the RT distributions from redundant signals trial types involving strong (loud and bright) bimodal stimuli. However, its fit was less accurate for trials with weak (soft and dim) bimodal stimuli, where predicted redundancy gains in choice RT exceeded the observed empirical values (see the left panel of Fig. 8). Despite this overestimation, these findings are intriguing given that the separate activation model was fit to the data without any free parameters. This approach may have been somewhat restrictive, as it relied solely on parameters estimated by fitting the Poisson-SLRT model to RT data from unimodal trial types. It is, therefore, possible that overfitting the Poisson-SLRT model to unimodal RT distributions may have contributed to a source of error that propagated into the fitting procedure for bimodal trials. This might warrant caution when interpreting these results. Nevertheless, we considered it necessary to fit the separate activation model and the coactive model based on parameter estimates obtained from unimodal trials to provide a fair comparison of the predictive capabilities of the two models.

We further observed that the coactive model provided a poor account of RT distributions from bimodal trials. This issue is clearly conveyed in Fig. 8 where the predicted RTs for both weak and strong bimodal stimuli are pronouncedly shifted to the left for all but the smallest quantiles relative to empirical data. Again, while it can be argued that our choice to fit the coactive model without any free parameters might have skewed the results, we find it unlikely that any other analysis approach would have favored the coactive model over the separate activation model. This is because the redundancy gains in RT predicted by the coactive model are so large that they simply cannot accommodate the relatively modest gains observed empirically. Moreover, the notion that predictions for error rates on bimodal trials arise naturally out of the separate activation model, whereas additional absorption barriers need to be calculated ad hoc for the coactive model based on empirical response probabilities, is another virtue that further strengthens the case against the coactivative model. Overall, although the race model inequality was violated for strong bimodal stimuli, quantitative model fits suggest that the championed coactive model account of redundancy gains in this task is untenable.

At this point, it needs to be underscored that the separate activation model and the coactive model only represent two possible instantiations of much larger classes of cognitive models. The validity of the separate activation model relies on the underlying assumptions of the horse race model (in particular, context independence). However, Mordkoff and Yantis (1991) have argued for intermediate types of cognitive architectures termed “interactive race models,” where processing occurs in parallel on channels A and B, but processing times on channel A can be affected by the presence or identity of a target on channel B, hence violating the assumption of context independence. Interactive racing can occur when probabilistic stimulus-stimulus or stimulus–response contingencies in the experimental design bias stimulus processing. In the present experiment, stimulus–stimulus contingencies were present, as auditory and visual intensity levels were perfectly correlated on bimodal trials. As discussed by Miller (2016), race models which violate context independence exhibit such flexibility that they can accommodate practically any distribution of RTs, rendering this model class fundamentally unfalsifiable. Conversely, the coactive model operates on the basis of an unweighted pooling mechanism of sensory evidence from auditory and visual channels. It is straightforward to see that an infinite set of coactive models can be construed by arbitrarily choosing the weights assigned to the outputs from visual and auditory channels. In sum, the broader issue of model mimicry (Mordkoff & Yantis, 1991; Townsend & Nozawa, 1997; Townsend, 1971) complicates the evaluation of coactive, race, and serial processing models. Certain reservations must therefore be acknowledged concerning the model-driven analyses and conclusions presented throughout this paper.

On a final note, the slowing of RTs for soft tones relative to loud tones warrants discussion. Typically, both simple and choice RTs are shorter for intense stimuli than for weak stimuli, a finding often attributed to faster sensory processing for more intense stimuli (Pins & Bonnet, 1996). It is therefore a violation of expectation that unimodal auditory RT data does not adhere to this established finding. One plausible explanation for the slower RTs to loud tones is that participants more readily identified the stimulus as soft rather than loud, leading to asymmetric error rates. In stochastic models of choice RT, such biased response tendencies are understood as criterion-setting effects, wherein the response threshold is lower for one stimulus category than the other, resulting in shorter decision times. At the decisional stage, this criterion-setting effect could counteract the faster sensory accrual typically observed for intense stimuli. However, this explanation is incomplete, as the unimodal visual condition presents a notable contrast: although error rates were higher for bright stimuli than for dim stimuli, bright targets still produced faster RTs.

The discrepant intensity effects for unimodal auditory and visual RTs could reflect differences in the magnitude, rather than the nature, of intensity and decision bias effects for the two types of stimuli. Alternatively, it may suggest fundamental differences between loudness identification and brightness identification processes that remain poorly understood. Further research is needed to clarify the underlying mechanisms driving these patterns. The Poisson-SLRT model addresses the dynamic interaction between stimulus intensity and decision bias. Intensity effects on sensory processing arise from the assumption that spike rates increase with stimulus intensity, while criterion-setting effects at the decisional stage are influenced by error rate asymmetries. These asymmetries, embedded within Eqs. 6 and 7, shift the absorption barriers to favor one response option over another. Hence, the model accommodates the potentially opposing effects of stimulus intensity and decision bias, as evident from commendable fit to unimodal RT distributions.

## Data Availability

Data and analysis code is available via https://osf.io/2yxvf/.

## Footnotes

1 Benchmarking data (line-by-line optosensor and microphone reads) are available via the Open Science Framework (OSF) at https://osf.io/sgz52.

2 https://osf.io/mdfwu/
